# Supplementary material for: Tiny Killers: First Record of Rhabdocoel Flatworms Feeding on Water Flea Embryos
Source: Ecol Evol. 2025 May 23;15(5):e71277. doi: 10.1002/ece3.71277 (PMC12100763; doi:10.1002/ece3.71277)
Supplement: Supplementary file 1 — Figure S1. The water well in which the observation of Strongylostoma simplex simplex flatworms preying on the water flea Daphnia magna was first observed (Luisenkirchhof II cemetery, Berlin, Germany). Figure S2. Flatworm infection by Strongylostoma simplex simplex (indicated with an arrow) in the smaller water flea species, Daphnia longispina , that co‐occurred in the sampling site with D. magna . Figure S3. Flatworm infection by Strongylostoma simplex simplex (indicated with arrows) in the water flea Daphnia magna . Note that flatworms seem to be attached to the water fleas’ovaries and/or midgut, potentially indicating feeding behaviour of the flatworms on tissues other than eggs. [file ECE3-15-e71277-s001.pdf]

SUPPLEMENTARY FIGURES

Tiny killers: first record of rhabdocoel flatworms feeding on water flea embryos

Nedim Tüzün[1], Nina Lemke[2], Yander L. Diez[3,4], Tom Artois[4], Marlies Monnens[4,5]

[1] Leibniz Institute of Freshwater Ecology and Inland Fisheries (IGB), 12587 Berlin, Germany

[2] Institut für Biologie/Zoologie, Freie Universität Berlin, 14195 Berlin, Germany

[3] Smithsonian Marine Station, 701 Seaway Drive, Fort Pierce, Florida 34949, United States

[4] Hasselt University, Centre for Environmental Sciences, 3590 Diepenbeek, Belgium

[5] Royal Belgian Institute of Natural Sciences, OD Taxonomy and Phylogeny, 1000 Brussels, Belgium

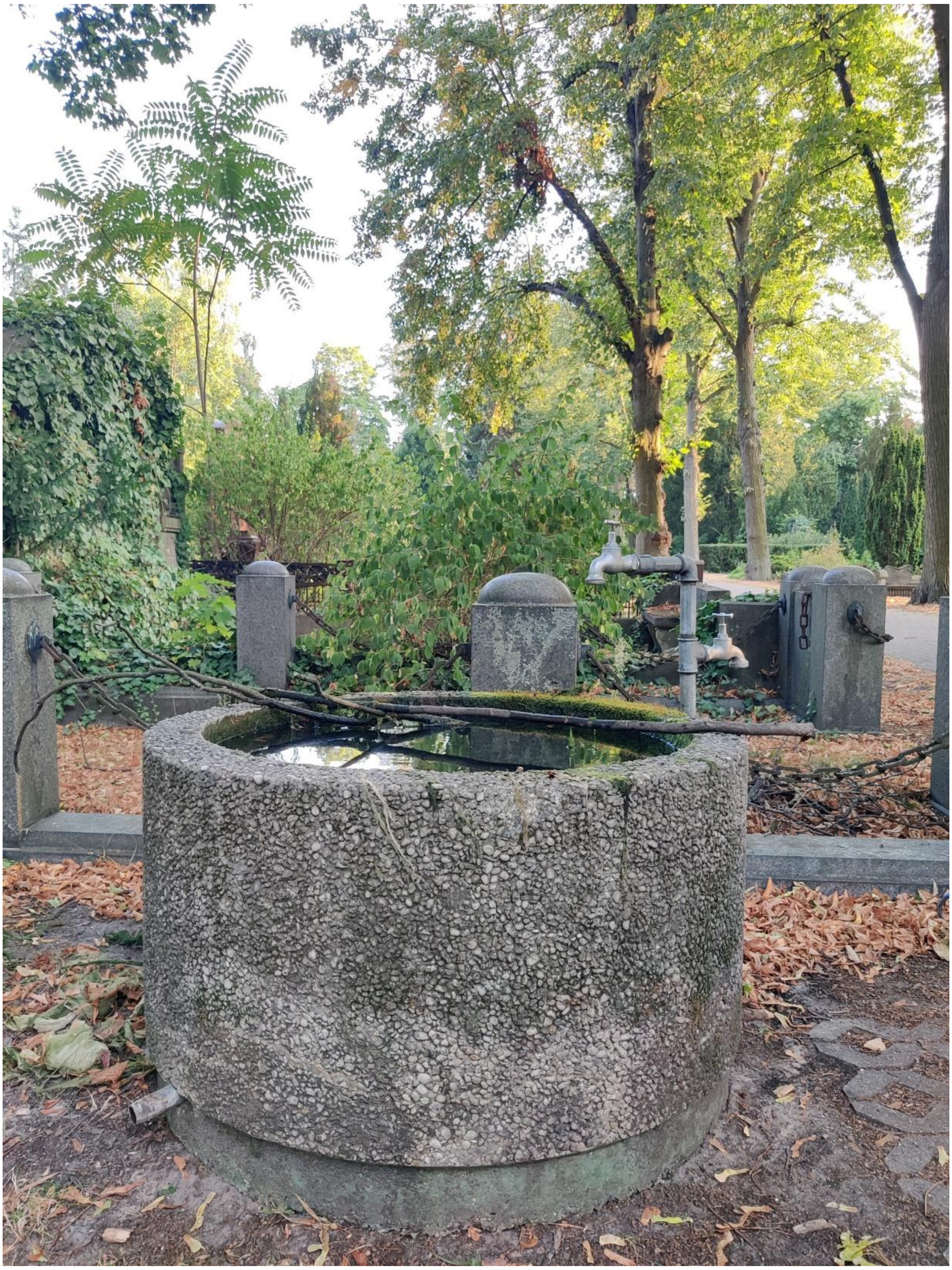

Figure S1. The water well in which the observation of *Strongylostoma simplex simplex* flatworms preying on the water flea *Daphnia magna* was first observed (Luisenkirchhof II cemetery, Berlin, Germany).

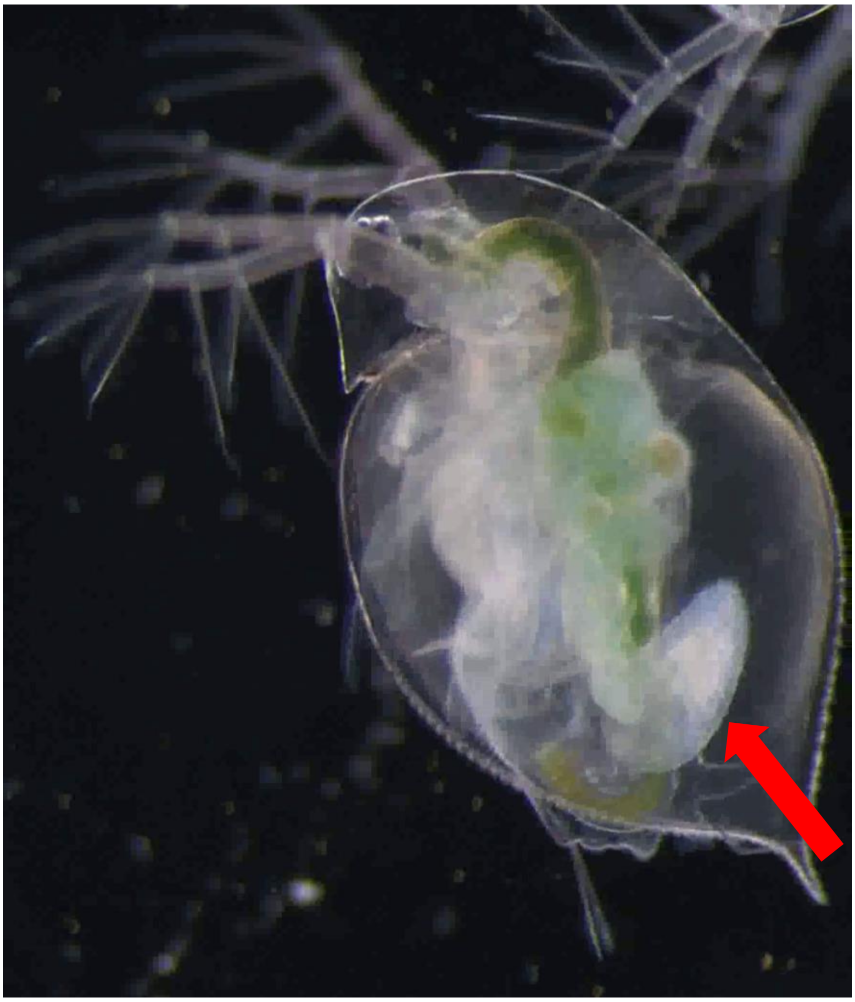

Figure S2. Flatworm infection by *Strongylostoma simplex simplex* (indicated with an arrow) in the smaller water flea species, *Daphnia longispina*, that co-occurred in the sampling site with *D. magna*.

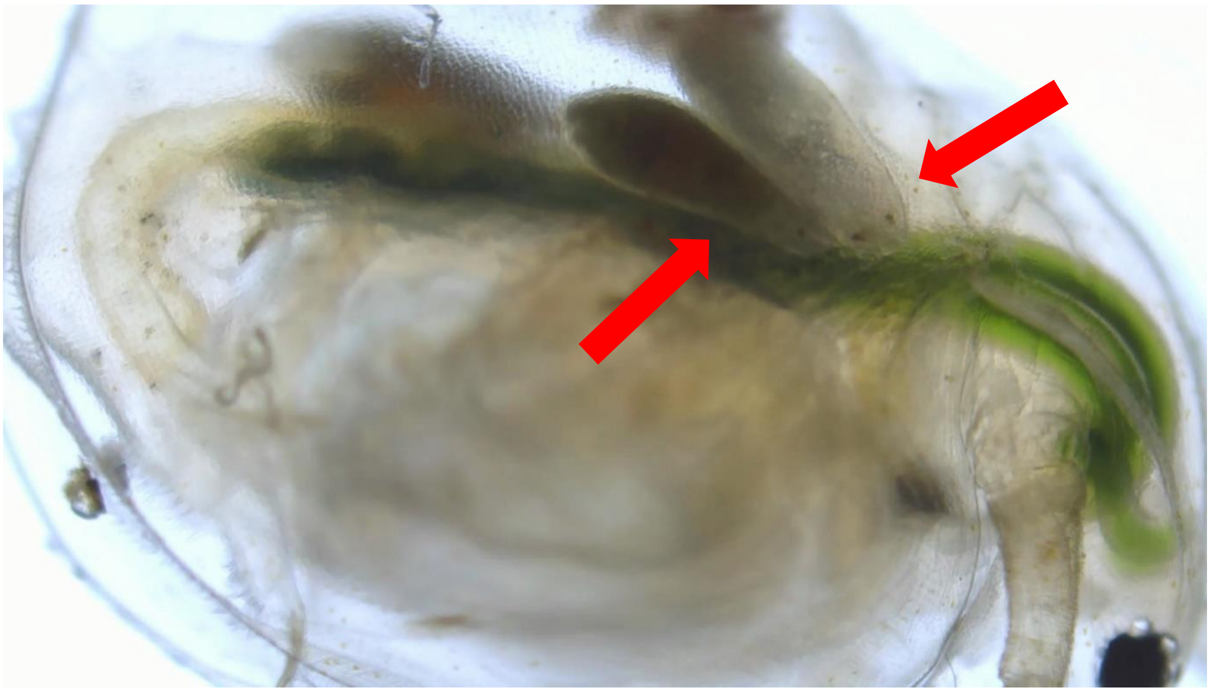

Figure S3. Flatworm infection by *Strongylostoma simplex simplex* (indicated with arrows) in the water flea *Daphnia magna*. Note that flatworms seem to be attached to the water fleas' ovaries and/or midgut, potentially indicating feeding behaviour of the flatworms on tissues other than eggs.
